# 4.4 Å Resolution Cryo-EM structure of human mTOR Complex 1

**DOI:** 10.1007/s13238-016-0346-6

**Published:** 2016-12-01

**Authors:** Huirong Yang, Jia Wang, Mengjie Liu, Xizi Chen, Min Huang, Dan Tan, Meng-Qiu Dong, Catherine C. L. Wong, Jiawei Wang, Yanhui Xu, Hong-Wei Wang

**Affiliations:** 1 Fudan University Shanghai Cancer Center, Institute of Biomedical Sciences, Shanghai Medical College of Fudan University, Shanghai, 200032 China; 2 Key Laboratory of Molecular Medicine, Ministry of Education, Department of Systems Biology for Medicine, School of Basic Medical Sciences, Shanghai Medical College of Fudan University, Shanghai, 200032 China; 3 State Key Laboratory of Genetic Engineering, Collaborative Innovation Center of Genetics and Development, School of Life Sciences, Fudan University, Shanghai, 200433 China; 4 Ministry of Education Key Laboratory of Protein Sciences, Tsinghua-Peking Joint Center for Life Sciences, Beijing Advanced Innovation Center for Structural Biology, School of Life Sciences, Tsinghua University, Beijing, 100084 China; 5 National Center for Protein Science, Shanghai Institute of Biochemistry and Cell Biology, Shanghai Institutes of Biological Sciences, Chinese Academy of Sciences, Shanghai, 200031 China; 6 National Institute of Biological Sciences, Beijing, 102206 China

**Keywords:** mTORC1, structure, cryo-electron microscopy

## Abstract

Mechanistic target of rapamycin (mTOR) complex 1 (mTORC1) integrates signals from growth factors, cellular energy levels, stress and amino acids to control cell growth and proliferation through regulating translation, autophagy and metabolism. Here we determined the cryo-electron microscopy structure of human mTORC1 at 4.4 Å resolution. The mTORC1 comprises a dimer of heterotrimer (mTOR-Raptor-mLST8) mediated by the mTOR protein. The complex adopts a hollow rhomboid shape with 2-fold symmetry. Notably, mTORC1 shows intrinsic conformational dynamics. Within the complex, the conserved N-terminal caspase-like domain of Raptor faces toward the catalytic cavity of the kinase domain of mTOR. Raptor shows no caspase activity and therefore may bind to TOS motif for substrate recognition. Structural analysis indicates that FKBP12-Rapamycin may generate steric hindrance for substrate entry to the catalytic cavity of mTORC1. The structure provides a basis to understand the assembly of mTORC1 and a framework to characterize the regulatory mechanism of mTORC1 pathway.

**Electronic supplementary material:**

The online version of this article (doi:10.1007/s13238-016-0346-6) contains supplementary material, which is available to authorized users.

## **INTRODUCTION**

Mechanistic target of rapamycin (mTOR) is a Ser/Thr kinase that belongs to the family of phosphoinositide-3-kinase-related kinases (PIKK) and is structurally and functionally conserved from yeast to mammals. mTOR exists in two distinct protein complexes: mTOR complex 1 (mTORC1) and mTOR complex 2 (mTORC2), which share two core components, the mTOR protein and the mammalian lethal with SEC13 protein 8 (mLST8, also known as GβL). mTORC1 contains a unique subunit, regulatory-associated protein of mTOR (Raptor), whereas mTORC2 is defined by rapamycin insensitive companion of mTOR (Rictor). Rapamycin inhibits mTORC1 by forming a complex with immunophilin FKBP12 (12 kDa FK506-binding protein) (Loewith et al., 2002; Sarbassov et al., 2004).

In response to multiple growth factors, energy status, and stress pathways, Tuberous Sclerosis Complex 1/2 (TSC1/2) complex serves as a negative regulator of mTORC1 and functions as a GTPase-activating protein (GAP) to inactivate the small GTPase Ras homolog Rheb (Garami et al., 2003; Inoki et al., 2003; Tee et al., 2003), which binds to and activates mTORC1. Nutrients promote the association of Raptor and Rag GTPases, which recruits mTORC1 to the surfaces of lysosomes and late endosomes for the activation by Rheb (Kim et al., 2008). Deregulation of mTORC1 has been found in many human diseases, especially in cancers (Dazert and Hall, 2011; Inoki et al., 2005; Tee and Blenis, 2005) and mTORC1 inhibitors have been clinically used for the treatment of organ transplantation and solid tumors (Benjamin et al., 2011). mTORC1 regulates cell growth primarily by phosphorylating a large number of proteins, including the eukaryotic initiation factor 4E (eIF4E) binding protein 1 (4EBP1) and p70-S6 Kinase 1 (S6K1) (Gingras et al., 1999; Holz et al., 2005; Holz and Blenis, 2005).

mTORC1 is one of the most important regulators to control cell growth and proliferation. The cellular function and dynamic regulation of mTORC1 have been extensively studied during the past decades. In contrast, the three-dimensional structure of mTORC1 remains largely unknown due to the technical difficulties in preparing the complex to homogeneity, as well as structural determination. The low-resolution (26 Å) cryo-electron microscopy (cryo-EM) structure of mTORC1 shows a two-fold symmetric dimer of complex formation (Yip et al., 2010). The crystal structure of the mTOR protein (deletion of N-terminal 1375 residues) in complex with mLST8 at 3.2 Å resolution was reported (Yang et al., 2013). The structure shows that the kinase domain adopts a canonical protein kinase conformation and provides a model for the inhibition of mTORC1 by Rapamycin-FKBP12. Recently, the cryo-EM structures of mTORC1 at 5.9 Å resolution (Aylett et al., 2016) and Tor-Lst8 from the thermotolerant yeast *Kluyveromyces marxianus* at 6 Å (Baretic et al., 2016) were reported, respectively. However, controversial conclusions were made for the topology of mTOR according to these studies. Here we report the cryo-EM structure of mTORC1 at 4.4 Å resolution. The higher resolution structure and biochemical analyses together provide a topological interpretation of human mTOR and relatively more accurate model for understanding the assembly and function of mTORC1.

## **RESULTS**

### **mTORC1 protein purification**

**Figure 1:**
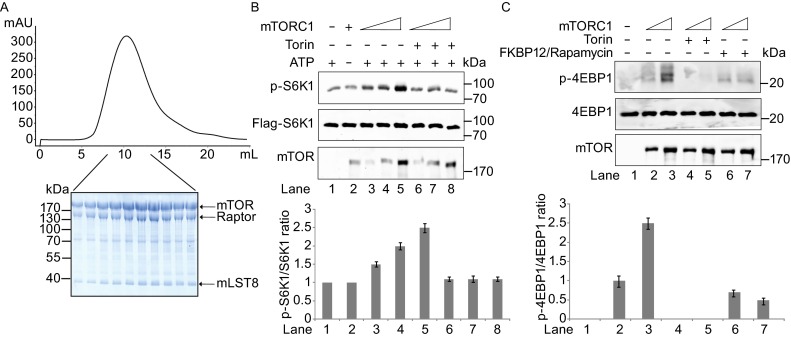
**Size exclusion chromatogram of the human mTORC1 and kinase activity**. (A) The gel-filtration was performed using a Superose 6 column (10/3004 GL, GE Healthcare). The peak fractions were subjected to SDS-PAGE and stained with Coomassie blue. (B and C) Phosphorylation of purified S6K1 (K100R) (B) and 4EBP1 (C) by mTORC1 in the presence or absence of Torin. The phosphorylation was detected by immunoblotting with antibodies targeting phospho-Thr-389 (top), Flag (middle), and mTOR (bottom) in (B), and antibodies targeting phospho-4EBP1 (top), 4EBP1 (middle), and mTOR (bottom) in (C). Below are the quantification of the immunoblots for B and C

To determine the cryo-EM structure of mTORC1, we purified the active ternary complex to homogeneity by the following procedures. The mTORC1 was transiently expressed with myc-mTOR, Flag-Raptor, Flag-mLST8 co-transfected into HEK293F cells in suspension culture. The complex was purified over an anti-Flag affinity resin, followed by ion exchange and gel filtration (Fig. 1A). The purified mTORC1 consists of mTOR, Raptor, and mLST8 in stoichiometry and exhibits kinase activity on S6K1 and 4EBP1, which can be inhibited by Torin1, a well-characterized ATP-competitive inhibitor of mTOR, and FKBP12-Rapamycin (Fig. 1B and 1C).

The mTORC1 was embedded in vitreous ice as mono-dispersed particles with multiple orientations, allowing us to perform single particle cryo-EM 3D reconstruction (Fig. S1A–C). Using a Titan Krios electron microscope equipped with a direct electron detector, we collected about 500,000 particle images of the mTORC1 and reconstructed the complex at an average resolution of 4.4 Å and the central portion was further refined to 4.0 Å resolution using a local mask (Methods, Supplementary information Fig. S1D–G, Table S1, and Movie 1). The Cryo-EM map showed well-defined secondary structural elements in the complex (Fig. S2), therefore allowing us to build the atomic models of the Raptor and mLST8 with unambiguous topology (Methods) except for the mTOR protein (see below).

### **The overall structure of mTORC1**

**Figure 2:**
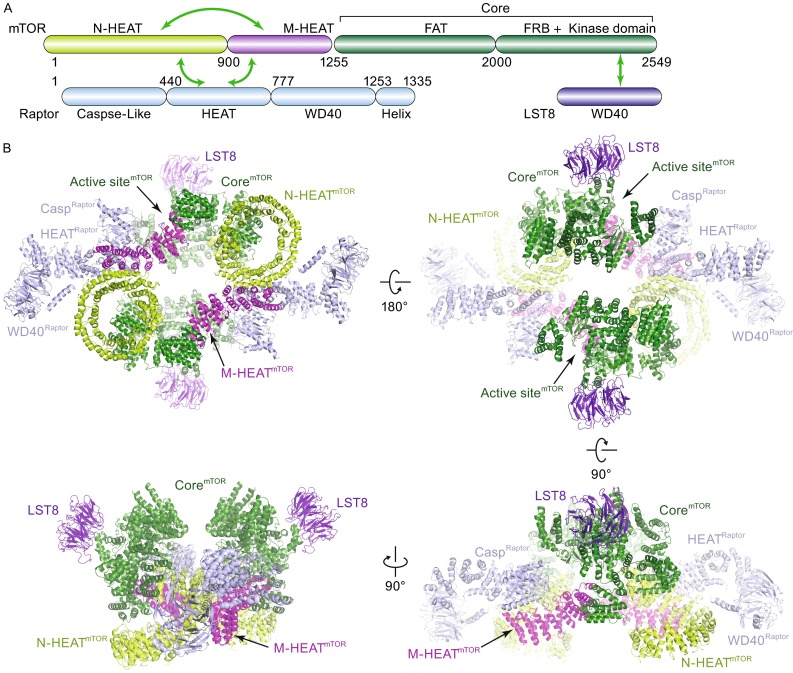
**Overall structure of human mTORC1**. (A) Colored coded domain architecture of the three essential components of human mTORC1. The same color scheme is used in all structure figures. The inter- and intramolecular interactions are indicated as arrows. B) Ribbon representation of mTORC1 structure in four different views. The proteins and domains are indicated. Casp^Raptor^ represents Caspase-like domain of Raptor

The mTORC1 architecture reveals a hollow rhomboid shape in the front view with overall dimensions of ~280 × 210 × 130 (Å^3^) (Fig. 2). The complex adopts a 2-fold symmetry with the central core primarily formed by two mTOR molecules. mLST8 is located on the distal convex along the short axis and protrudes out of the rhomboid architecture. Each Raptor binds to two mTOR molecules and extends towards the distal convex along the long axis of the rhomboid with the WD40 repeats domain located on the apical side. A central hole is formed within the mTOR dimer. From a side view, the N-terminal super-helical α-solenoids of mTOR and Raptor are located on one side of the rhomboid whereas the kinase domain of mTOR and the associated mLST8 are on the other side (lower panels in Fig. 2B). The kinase domain is close to mLST8 and the Caspase-like domain of Raptor and the open catalytic cavity faces towards outside of the central core region. Our cryo-EM analysis revealed a strong heterogeneous conformation of the dimeric mTORC1 with the distance between edges of two kinase domains ranging from 9 Å to 29 Å even after the mTORC1 cryo-EM sample was generated with mild glutaraldehyde gradient fixation (Fig. S3), which explains the difficulty in improving the resolution of mTORC1 reconstruction.

The C-terminal region of mTOR protein forms a compact core domain (designated Core), in which a “C”-shaped α-solenoid (FAT) wraps around the kinase domain from which the FRB domain protrudes out (Fig. 2B). The N-terminus of mTOR protein adopts a spiral (~1.3-turn) right-hand super-helix comprising of 16 HEAT repeat (designated N-HEAT). The middle region adopts extended HEAT repeat (designated M-HEAT) bridging the N-HEAT and the Core domain of mTOR. Intriguingly, no direct contact was observed between the N-HEAT and M-HEAT nearby the Core domain. Furthermore, while the M-HEAT’s two ends are well defined in the EM map, no density was observed to connect the two ends to either the N-HEAT or the Core regions even at lower threshold. Therefore, the mTOR protein adopts a peculiar disconnected/branched domain architecture within the mTORC1 so that the mTOR protein’s topology is difficult to be unambiguously determined solely from the 4.4 Å resolution reconstruction.

### **Topology of mTOR protein**

**Figure 3:**
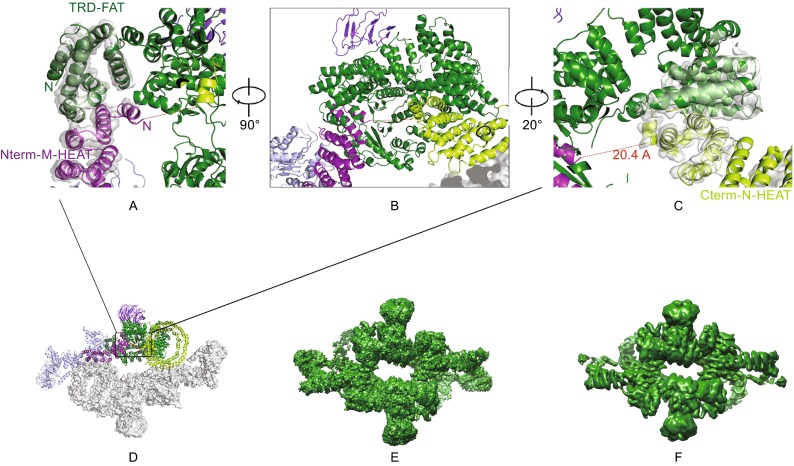
**The cryo-EM electron potential maps for the interfaces between M-HEAT and Core and between N-HEAT and M-HEAT (D) contoured at 5 sigma level. (A–C) is the different zoom-in view**. The putative linkage between N-HEAT and M-HEAT is depicted as the red dot line, which is measured to be 20.4 Å. Because of the high quality of the cryo-EM map, it is clear that the density making up the M-HEAT region does not directly join with the FAT domain. (E and F) Comparison of cryo-EM map from this study at 4.4 Å (E) and previous study at 5.9 Å (F) resolution, respectively. Both of the maps are displayed in Chimera at the same threshold after normalization

We sought to use a combination of bioinformatics, crosslinking mass spectrometry and structural biology approaches to determine the topology of mTOR protein. The C-terminal regions could be clearly defined according to the previous crystal structure (Yang et al., 2013). However, the topology of the N-HEAT and M-HEAT in mTOR protein is hard to define. Opposite orientations of N-HEAT and M-HEAT from two mTOR molecules can generate eight possible topology models for the mTOR dimer. Taking account of the proximity likelihood among N-HEAT, M-HEAT and the Core domain, there are four possible models worth further consideration (Fig. S4). The relatively good quality and high resolution of our potential map in the central portion of the reconstruction revealed clearly that the density corresponding to the M-HEAT region does not directly join with TRD subdomain (numbering according to the crystal structure of Core^mTOR^) (Yang et al., 2013) of FAT domain (Fig. 3, Movie 2). A short segment of helix from M-HEAT extends in the opposite direction away from the N-terminus of TRD, with a straightaway distance of 35 Å between the two termini. Interestingly, one terminus of the N-HEAT domain also extrudes out a short helix from its HEAT repeat motif, which faces toward the M-HEAT domain (Fig. 3, Movie 2). The distance between the above two short helix segments is approximately 20 Å, agreeing with the secondary structural prediction that there are ~10 residues as a flexible loop between the N-HEAT and M-HEAT (Fig. S5). This supports a topology of model I for the mTOR protein in the complex. This model is further supported by crosslinking mass spectrometry analysis of the mTORC1, with the most confident lysine crosslinking pairs between the N-HEAT and the Core region, between the M-HEAT and the Core region, and between the M-HEAT and the caspase-like domain of Raptor (Fig. S6). Notably, this topology (model I) was interpreted differently in the cryo-EM structure of mTORC1 at 5.9 Å resolution (Aylett et al., 2016), but in agreement with the topological interpretation of Tor-Lst8 structure from the thermotolerant yeast *Kluyveromyces marxianus* (Baretic et al., 2016).

### **Interaction of mTORC1 subunit**

**Figure 4:**
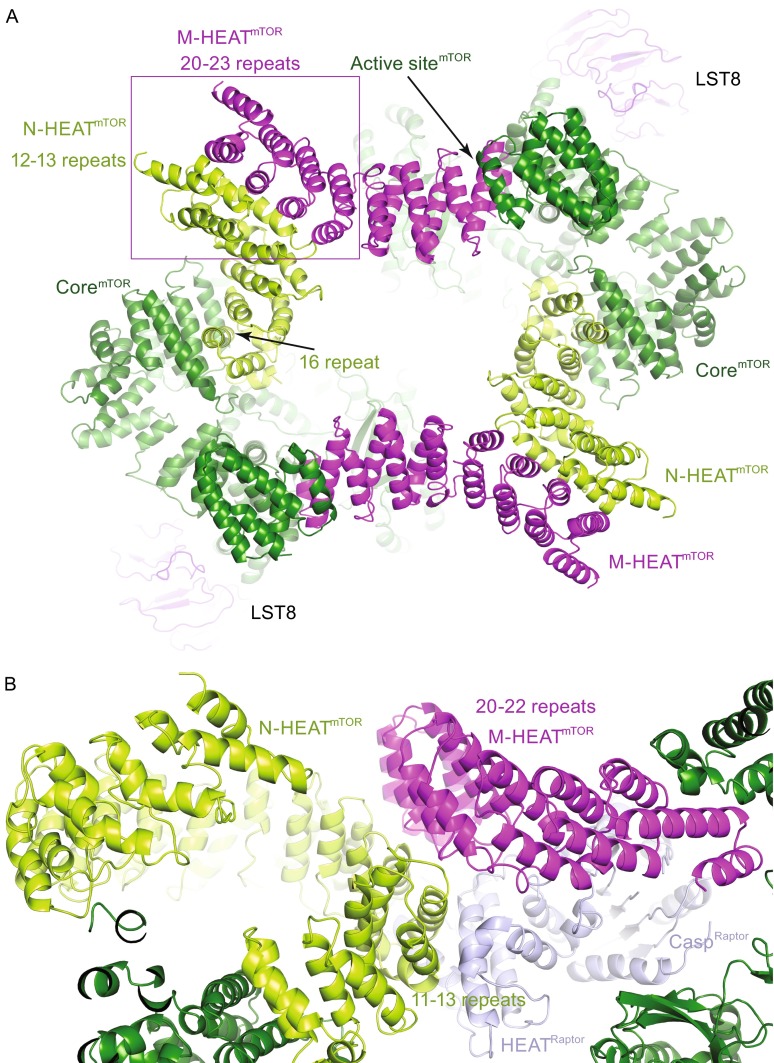
**Intramolecular and intermolecular interactions within mTORC1**. (A) Closed-up view of the dimer formation of mTORC1. Ribbon representation of the mTORC1 is shown. The mTORC1 dimer is formed through the intermolecular interaction between the M-HEAT and the Core and that between the N-HEAT and the Core. (B) Closed-up view of the interaction between Raptor and mTOR. Raptor binds to the ridge region of the M-HEAT repeats in one mTOR and the convex side of the N-HEAT region in the other mTOR

We therefore used the model I hereafter for structural analyses of inter- and intramolecular interactions within the mTORC1 (Fig. 4). Within the N-HEAT region, the HEAT repeats 12–13 are involved in the interaction with Raptor and the M-HEAT region. The HEAT repeat 16 binds to three parallel α-helices of FAT domain of the Core region. Other parts of the N-HEAT region have no inter- or intramolecular interactions, suggesting that N-HEAT tends to be dynamic, in consistent with its relatively weak density in the 3D reconstruction. Similar phenomenon has been observed in other proteins such as the flexible HEAT repeats of PP2A complex (Xu et al., 2006). The conformational flexibility of the N-HEAT region may provide significant functional implications, for example, adopting different conformations for the interactions with various mTOR regulators (Kim et al., 2008; Wullschleger et al., 2006; Zoncu et al., 2011).

The M-HEAT region contains 7 HEAT repeats (HEAT repeats 17–23) and adopts extended conformation. The dimerization of mTOR protein in the complex is mediated by the inter-molecular interaction between the N-HEAT region from one mTOR and the M-HEAT region from the other one (Fig. 4A). This interaction is through close proximity between the HEAT repeats 20–23 of the M-HEAT region and the HEAT repeats 12–13 of the N-HEAT region. The formation of this interaction does not seem to need other proteins but is stabilized by Raptor, which binds to the ridge region of the M-HEAT repeats 20–22 in one mTOR and the convex side of the N-HEAT repeats 11–13 in the other mTOR (Fig. 4B). To test Raptor’s role in the dimerization of mTOR protein, we purified mTOR-mLST8 complex without Raptor. Gel-filtration analysis indicates that mTOR-mLST8 is sufficient to dimerize (Fig. S7). Thus, mTOR-mLST8 can assemble into dimer of heterodimer, which serves as the core for mTOR holoenzyme (mTORC1 or mTORC2) assembly, as well as a scaffold for association with various binding proteins. We propose that mTOR-mLST8 adopts similar fold in mTORC1 and mTORC2 complexes. The mTOR surface for Raptor interaction may also be involved in Rictor association because Raptor and Rictor are mutually exclusive for mTOR complex formation (Loewith et al., 2002; Sarbassov et al., 2004).

### **Structure and implication of RNC domain of Raptor**

**Figure 5:**
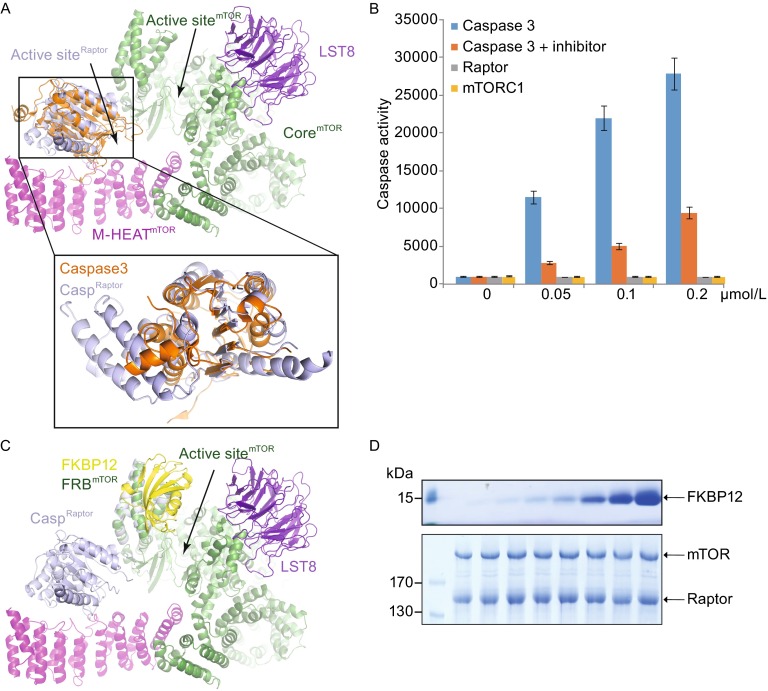
**Caspase-like domain of Raptor and FKBP12-mTORC1 interaction**. (A) Superimposition of caspase-3 and Raptor in mTORC1 structures. Two structures are shown in ribbon representations with unnecessary regions omitted. Caspase-3 is colored in orange. The active site of kinase domain of mTOR and the active site of caspase-like domain of Raptor are indicated, respectively. Shown below is the closed-up view of the structural comparison between caspase-3 and caspase-like domain of Raptor. (B) Equal amount of purified caspase-3 and Raptor were incubated with 20 µmol/L Ac-DEVD-AMC in the presence or absence of caspase inhibitor Z-VAD-FMK at 37°C for 60 min. The activities were measured using a Spectrafluor Fluorescence Plate Reader with excitation at 400 nm and emission at 505 nm. Error bars, s.d. for triplicate experiments. (C) Superimposition of FRB^mTOR^-FKBP and mTORC1 structures is shown in ribbon representations. (D) Effect of FKBP12-Rapamycin for mTORC1 assembly. Increased amount of FKBP12-Rapamycin incubated with mTORC1 (Flag-Raptor) immobilized on the Flag resin. Bound proteins were subjected to SDS-PAGE and stained with Coomassie blue

The mTORC1 structure shows that Raptor contains a Raptor N-terminal conserved (RNC) domain, followed by HEAT repeats and a C-terminal WD40 repeats. The RNC domain adopts a compact fold with a six-stranded β-sheet stabilized by three α-helices on each side. In support of previous prediction (Ginalski et al., 2004), the RNC domain shows similar overall structure to that of caspase family proteins (Fig. 5A). Although the catalytic residues (H153, C196) of Raptor are conserved, no caspase activity was detected using Ac-DEVD-AMC as the substrate for either the Raptor alone or mTORC1 (Fig. 5B).

### **Proposed mechanism of FKBP12-Rapamycin inhibition on mTORC1 activity**

It has been well characterized that FKBP12-Rapamycin binds to mTORC1 and inhibits its kinase activity. Structural comparison of mTORC1 with FRB-FKBP12 (Choi et al., 1996) shows that upon binding to FRB, FKBP12 would be in close proximity to kinase domain of mTOR and therefore lead to steric hindrance for substrate entry into the catalytic cavity of mTORC1 (Fig. 5C). Notably, structural superimposition shows that FKBP12 has no other direct contact with mTORC1 besides FKBP-FRB interface. There is still ample space for substrate association, which explains the relatively weak inhibition by FKBP12-Rapamycin compared to the ATP-competitive inhibitor Torin1. To test whether FKBP12 association disrupts interaction between mTOR and Raptor (Kim et al., 2002), we performed an in vitro assay using purified mTORC1 (Flag-Raptor) and increasing amount of FKBP12-Rapamycin (Fig. 5D). In support of the structural analysis, FKBP12-Rapamycin does not disrupt mTORC1 integrity.

## **DISCUSSION**

In this study, we report the cryo-EM structure of mTORC1 at 4.4 Å resolution, the highest resolution for human mTORC1 up to now. According to the biochemical and structural analyses, we proposed a most likely correct topological model for mTOR within the complex. The structure provides a structural basis for understanding the complex assembly of mTORC1 and the regulatory mechanism of mTORC1 by its binding partners and those yet to be identified.

First, we provided four possible models and used several ways to determine the topology of mTOR protein. Our result support the topology of model I, which is in accordance with Tor-Lst8 structure from yeast (Baretic et al., 2016), while is not consistent with mTORC1 at 5.9 Å resolution (Aylett et al. 2016). Although further high-resolution structural analysis should be performed, we here propose that model I is more likely to represent correct topology of mTOR under current understanding (Fig. S4).

Second, from structural analysis, the RNC domain of Raptor bind to the M-HEAT and N-HEAT repeats of mTOR (Fig. 4B). It has been reported that Ser 863, Ser 859 phosphorylation of Raptor by several kinases in response to different conditions, leading to a decrease of mTORC1 activity (Dunlop et al. 2014; Stretton et al., 2015; Yuan et al. 2015) because of reduced interaction between mTOR and Raptor (Stretton et al., 2015). However, structural analysis shows that Ser 859 of Raptor is located within the WD40 repeat domain and would have no direct contact with mTOR. Thus, the structure of mTORC1 also provides a framework to reconsider previous mechanistic explanation of biochemical observations of mTORC1 pathway.

Third, although the RNC domain of Raptor is similar with caspase family proteins from overall structure, we test that Raptor has no caspase activity (Fig. 5B). Previous studies have shown that Raptor binds to and recruits mTORC1 substrate proteins through recognizing TOS motifs, FDIDL in S6K1 and FEMDI in 4EBP1, both containing conserved aspartate residues for caspase cleavage. Therefore, the caspase-like domain may serve as a module to recognize and recruit specific substrates for kinase processing. Consistently, the catalytic site of the caspase-like domain of Raptor faces toward the catalytic cavity of the kinase domain of mTOR.

Fourth, our structural analysis and experiment result show that FKBP12-Rapamycin does not disrupt mTORC1 integrity as previously discussed (Kim et al., 2002). In fact, it provides steric hindrance for substrate entry into the catalytic cavity of mTORC1. In addition, higher resolution structure of mTORC1 is needed to illustrate the detailed information of the complex.

## **MATERIALS AND METHODS**

### **Reagents**

Antibodies against phospho-Thr-389 S6K (9205), 4EBP1 (9652), phos-4EBP1 (9456) and mTOR (2972) were from Cell Signaling Technology; horseradish peroxidase-labeled anti-mouse and anti-rabbit secondary antibodies were from AbMart; FLAG M2-agarose was from Sigma; Ni-NTA resin, Mono Q, Superdex75 (10/300 GL) and Superose 6 (10/300 GL) were from GE Healthcare; Rapamycin and Z-VAD-FMK were from Selleckchem; Torin1 was kindly provided by Dr. Haixin Yuan from IBS, Fudan University; Polyethylenimine (PEI) was from Polysciences (23966); 293F medium was from Sino Biological Inc.

### **Protein expression and purification**

To produce soluble mTORC1 protein, the ORFs of human mTOR, raptor, and mLST8 were sub-cloned into modified pCAG vectors. The three plasmids were co-transfected to 293F cells using PEI. After culture at 37°C for 3 days, cells were collected and lysed in 50 mmol/L HEPES, pH 7.4, 150 mmol/L NaCl, 0.4% CHAPS and 3 mmol/L DTT at 4°C for 30 min, and the insoluble fraction was removed by centrifugation at 15,000 rpm for 30 min. Supernatants were incubated with FLAG-M2 monoclonal antibody-agarose for 1 h and unbound proteins were extensively washed away. The fusion proteins (FLAG-tagged raptor, FLAG-tagged mLST8 and Myc-tagged mTOR) were digested using PreScission protease overnight and the eluted proteins were further purified using ion exchange and gel filtration chromatography. The peak fractions were pooled for biochemical and structural analyses.

### ***In vitro*kinase assays**

The in vitro kinase assays were performed in the buffer containing 25 mmol/L HEPES, pH 7.4, 100 mmol/L NaCl, 10 mmol/L MgCl_2_, 2 mmol/L DTT and 0.5 mmol/L ATP for 30 min at 30°C. Reactions were terminated by the addition of SDS sample loading buffer and boiling for 5 min. Samples were subsequently analyzed by SDS-PAGE and immunoblotting.

#### *Caspase activity assay*

The purified human caspase-3 and Raptor were respectively incubated with 20 µmol\L Ac-DEVD-AMC with or without caspase inhibitor Z-VAD-FMK at 37°C for 60 min using a Spectrafluor Fluorescence Plate Reader with excitation at 400 nm and emission at 505 nm.

### **Purification of FKBP12 and in vitro pull-down**

FKBP12 protein was purified from *E. coli* BL21(DE3) cells transformed with modified pGEX-6P-1 vector containing the ORF of human FKBP12. GST-FKBP12 was induced at 16 °C overnight with 0.1 mmol/L IPTG. The cells were harvested and disrupted with buffer containing 25 mmol/L Tris, pH 8.0, 150 mmol/L NaCl, 5 mmol/L imidazole. The fusion proteins were purified using Ni-NTA resin and digested by PreScission enzyme. The eluted proteins were purified using ion exchange and gel filtration. Equal molar FKBP12 and rapamycin were incubated with Flag-Raptor/mTOR for 30 min. The unbound proteins were washed away and the bound proteins were subjected to SDS-PAGE and stained by Coomassie blue.

### **Cross-linking and Mass Spectrometry analysis**

The purified mTORC1 (0.8 µg/µL) was cross-linked using disuccinimidyl suberate (DSS) at a 1:150 molar ratio at room temperature for 20 min. The reaction was terminated by 20 mmol/L ammonium bicarbonate. The proteins were precipitated with cooled acetone and lyophilized. The pellet was dissolved in 8 mol/L urea, 100 mmol/L Tris, pH 8.5, followed by TCEP reduction, iodoacetamide alkylation, and trypsin digestion. Trypsin (Promega) digestion was quenched by 5% formic acid. Tryptic peptides were desalted with Pierce C18 spin column (Thermo Fisher) and separated in a Proxeon EASY-nLC liquid chromatography system by applying a step-wise gradient of 0–85% acetonitrile (ACN) in 0.1% formic acid. Peptides eluted from the LC column were directly electrosprayed into the mass spectrometer with a distal 2 kV spray voltage. Data-dependent tandem mass spectrometry (MS/MS) analysis was performed on Thermo Q-Exactive instrument in a 60-minute gradient. Raw data was processed with pLink software (Ellisen et al. 2002).

### **Cryo-electron microscopy**

For cryo-grid preparation, aliquots of 3.5 µL of purified mTOR1 complex at a concentration of ~1.5 mg/mL were applied to glow-discharged holey carbon grids (Quantifoil Cu, R1.2/1.3, 400 mesh). The grids were blotted for 2.5 s and flash-plunged into liquid ethane pre-cooled in liquid nitrogen using an FEI Vitrobot mark IV operated at 22°C and 100% humidity. Data collection was performed on an FEI Titan Krios equipped with Gatan K2 Summit electron counting camera. Images were recorded in the super-resolution mode using UCSF-Image4 (Wang et al., 2015) at a nominal magnification of 22,500, which corresponds to a final pixel size of 1.306 Å by binning 2 of the original micrographs. For each image stack, a total dose of about 50 electrons per Å^2^ at the specimen were equally fractioned into 32 frames with a total exposure time of 8 s. Defocus values ranged from −1.5 to −2.5 μm.

### **Image processing**

For cryo-EM data sets, beam-induced motion correction was performed as previously described (Li et al., 2013). Micrographs inspection, automatic particle picking, 2D, 3D classification and refinement were performed within RELION 1.4 (Scheres, 2012) and the contrast transfer function parameters were estimated using CTFFIND3 (Mindell and Grigorieff, 2003). About 2000 manually picked particles were used to generate the templates for particle auto-picking. Totally 486,584 particles were auto-picked from 2997 micrographs for further processing. After one round of reference-free 2D classification, 387,806 particles were then subjected to 3D classification using the previously reconstructed 3D map (EMDB:5197) at 20 Å resolution as the initial model. Two rounds of 3D classification were performed to remove the obvious bad particles in the first round and accumulate the most homogeneous population of particles in the second round. A final dataset of 115,039 most homogeneous particles were subjected for final refinement to generate a 4.4 Å resolution map through the process of auto-refinement with C2 symmetry imposed (gold-standard FSC 0.143 criteria) (Chen et al., 2013), particle polishing, and post-processing with auto-mask and the manual-bfactor option (B-factor of −60 Å^2^) in RELION. In a final step, a soft mask was imposed on the rigid central portion of the map to improve the density quality by continuing run of the auto-refinement in RELION. This yielded a final 3D map of the central portion at an average of 4.0 Å resolution (corrected gold-standard FSC at 0.143).

### **Model building into the cryo-EM map**

The crystal structure of mTOR (1385–2549aa)/mLST8 (PDB ID: 4JSN) (Yang et al., 2013) was docked into the mTORC1 cryo-EM map using EMfit (Rossmann et al., 2001) while maintaining the two-fold symmetry. The rest parts of mTOR, including N-HEAT and M-HEAT domains, were built by placing the ideal alpha-helices into the density using COOT (Emsley et al., 2010). The caspase-like domain and WD40 domain of Raptor were built by docking the crystal structures of caspase-9 (PDB ID: 2AR9) and WD40 domain (PDB ID: 4J87) into the density. The HEAT repeat domain of Raptor was built manually in COOT (Emsley et al., 2010). The coordinates of the final structure were refined in the real space using phenix.real_space_refine (Adams et al., 2010). Model validation was performed with PROCHECK (Laskowski et al., 1993) and the WHATCHECK routine of WHAT IF (Vriend, 1990).

## Electronic supplementary material

Below is the link to the electronic supplementary material.

Supplementary material 1 (MOV 6479 kb)

Supplementary material 2 (MOV 6059 kb)

Supplementary material 3 (PDF 2916 kb)
