# Supplementary material for: 4.4 Å Resolution Cryo-EM structure of human mTOR Complex 1
Source: Protein Cell. 2016 Dec 1;7(12):878–87. doi: 10.1007/s13238-016-0346-6 (PMC5205667; doi:10.1007/s13238-016-0346-6)
Supplement: Supplementary file 3 — Supplementary material 3 (PDF 2916 kb) [file 13238_2016_346_MOESM3_ESM.pdf]

	mTORC1 complex
CC across whole map volume	80.73%
RMS bonds (Å)	0.011
RMS angles (°)	1.786
Ramachandran statistics (%)	
Most favored	80.7
Additional allowed	16.9
Generously allowed	1.9
Disallowed	0.5

Table S1: Statistics after refinement of atomic coordinates for mTORC1 complex.

Yang_Fig S1

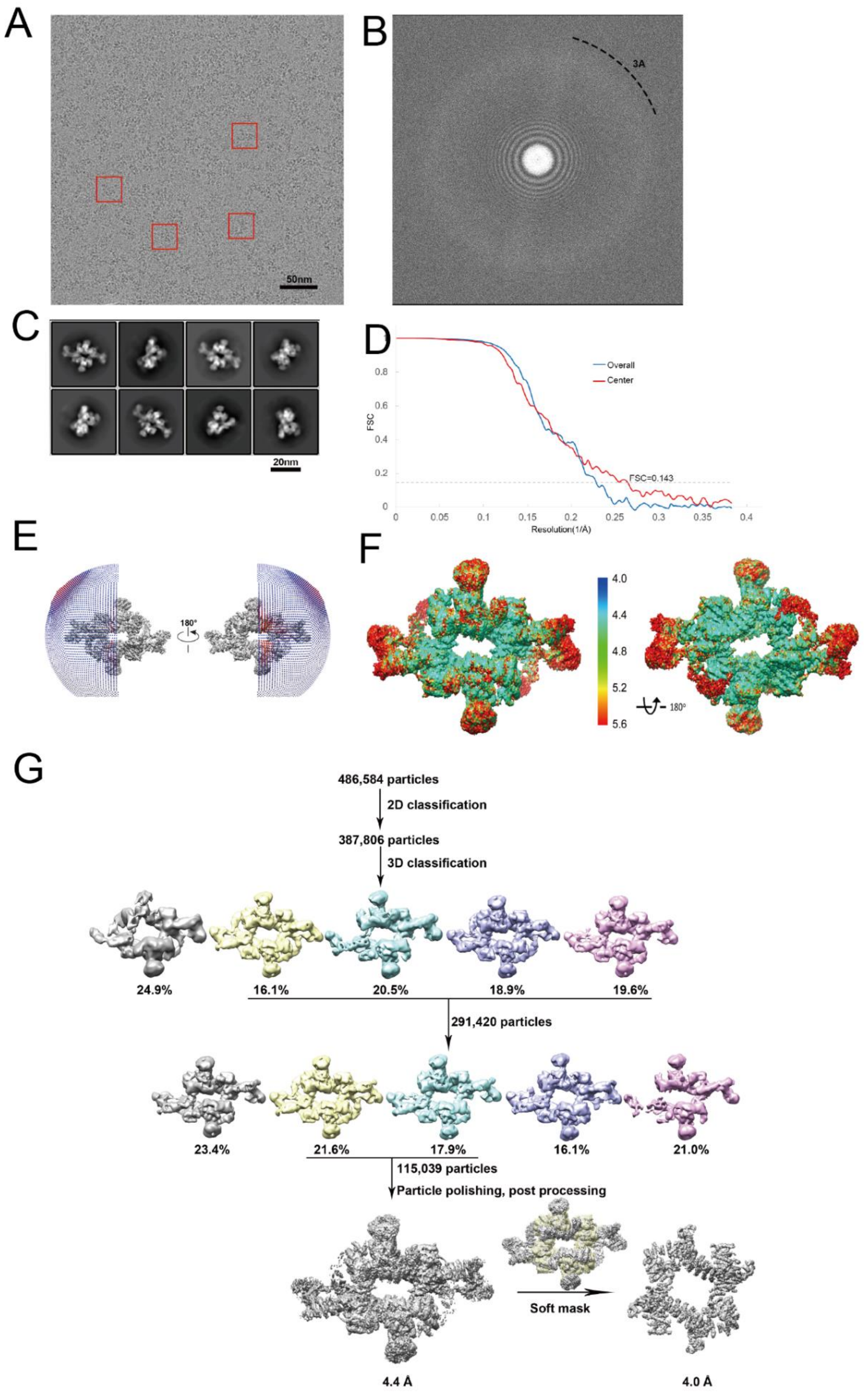
A
50nm
B
3A
C
20nm
D
Overall
Center
FSC
FSC=0.143
Resolution(1/Å)
E
180°
F
4.0
4.4
4.8
5.2
5.6
180°
G
486,584 particles
2D classification
387,806 particles
3D classification
24.9%
16.1%
20.5%
18.9%
19.6%
291,420 particles
23.4%
21.6%
17.9%
16.1%
21.0%
115,039 particles
Particle polishing, post processing
Soft mask
4.4 Å
4.0 Å

Figure S1: (A-B) A representative raw micrograph of cryo-EM mTORC1 (A) and its Fourier transforms (B). (C) Representative 2D Class average of Cryo-EM mTORC1 particles. (D)Angular distribution of cryo-EM mTORC1 used for final structuralrefinement. (E) FSC curves for the final 3D density map after B-factor sharpening before (red) and after (blue) soft mask application on the central part of mTORC1 in RELION (gold-standard FSC) was corrected by radon phase substitution. (F) Local resolution map of mTORC1 calculated by ResMap. (G) A flow-chart of cryo-EM data processing.

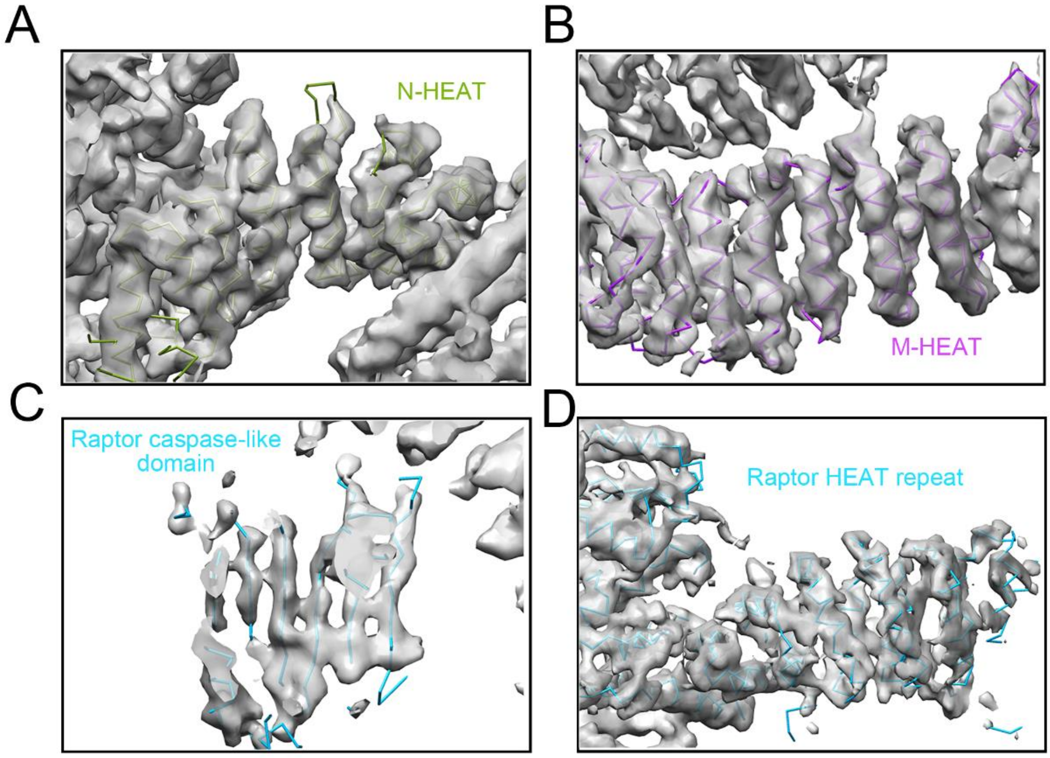

Figure S2: (A-D) A representative cryo-EM densities of the N-HEAT region, M-HEAT region, Raptor caspase-like domain,2 and Raptor HEAT repeat, respectively.

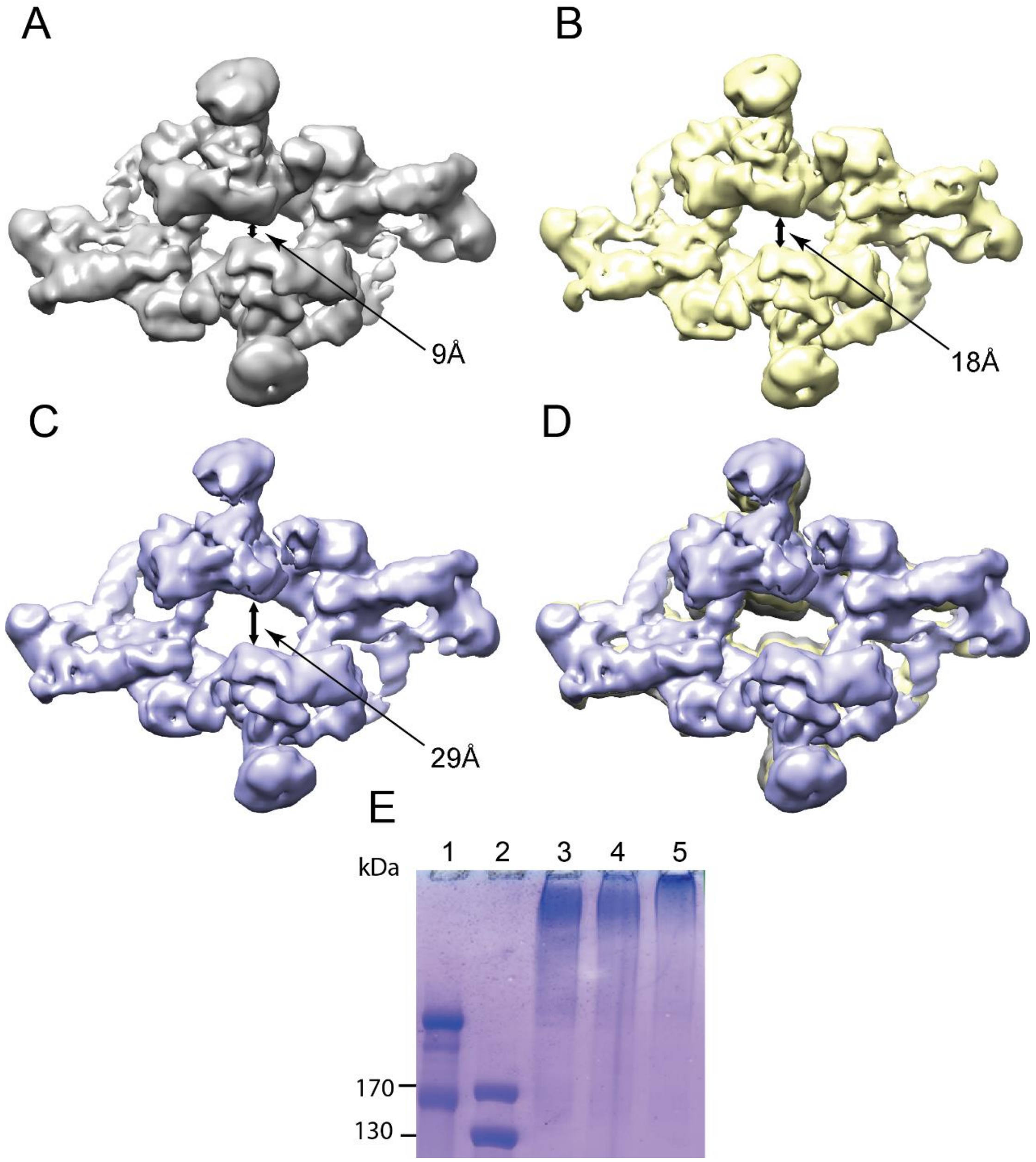

Figure S3: (A-C) Three representative classes of mTORC1 during 3D classification. The variation of the indicated di3stance shows conformational dynamics within mTORC1. (D) The superimposition of the three classes of mTORC1. (E) mTORC1 were subjected to SDS-PAGE and stained with Coomassie blue. Lane 1: mTORC1 after gel filtration, lane 2: protein marker; lane3-5: mTORC1 was treated with mild glutaraldehyde gradient fixation. Lane 3 sample was used for cryo-EM.

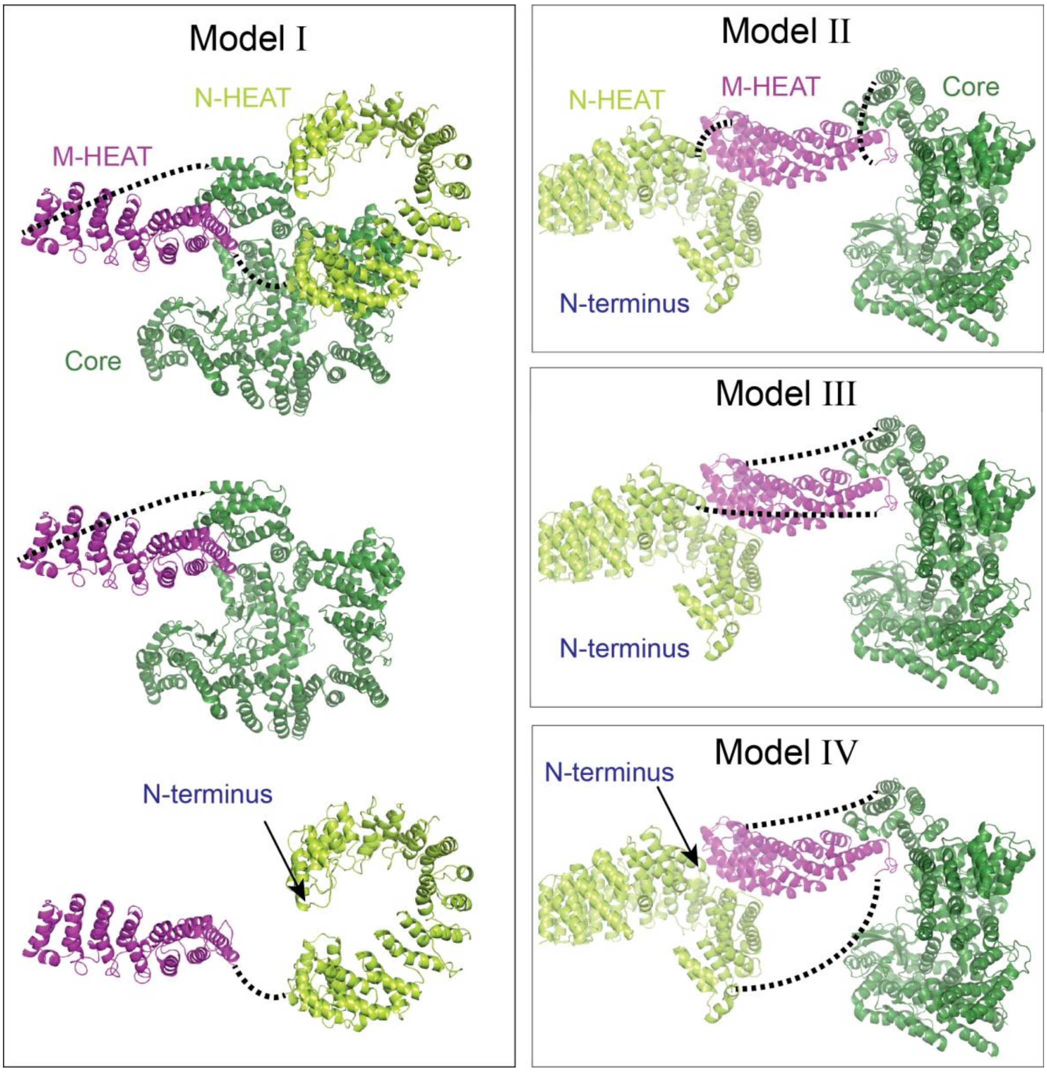

Figure S4: Monomeric mTORC1 is shown as ribbon representation for simplicity. The potential density for residues connecting N-HEAT and M-HEAT, and that connecting M-HEAT and Core, are invisible in 4.4 Å resolution map. We therefore built four possible structural models as indicated.

Yang_Fig S5

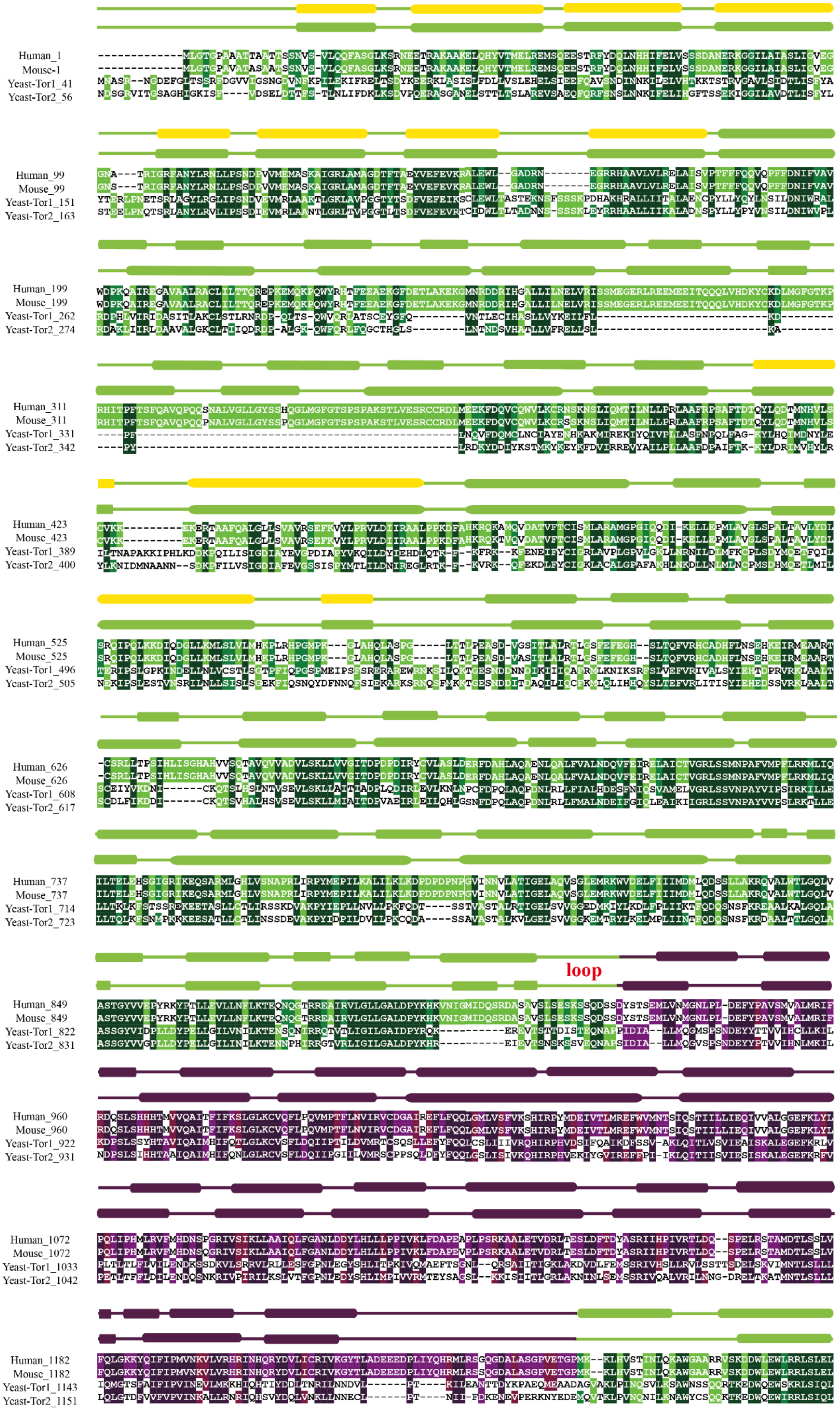
Human_1
Mouse-1
Yeast-Tor1_41
Yeast-Tor2_56
Human_99
Mouse_99
Yeast-Tor1_151
Yeast-Tor2_163
Human_199
Mouse_199
Yeast-Tor1_262
Yeast-Tor2_274
Human_311
Mouse_311
Yeast-Tor1_331
Yeast-Tor2_342
Human_423
Mouse_423
Yeast-Tor1_389
Yeast-Tor2_400
Human_525
Mouse_525
Yeast-Tor1_496
Yeast-Tor2_505
Human_626
Mouse_626
Yeast-Tor1_608
Yeast-Tor2_617
Human_737
Mouse_737
Yeast-Tor1_714
Yeast-Tor2_723
loop
Human_849
Mouse_849
Yeast-Tor1_822
Yeast-Tor2_831
Human_960
Mouse_960
Yeast-Tor1_922
Yeast-Tor2_931
Human_1072
Mouse_1072
Yeast-Tor1_1033
Yeast-Tor2_1042
Human_1182
Mouse_1182
Yeast-Tor1_1143
Yeast-Tor2_1151

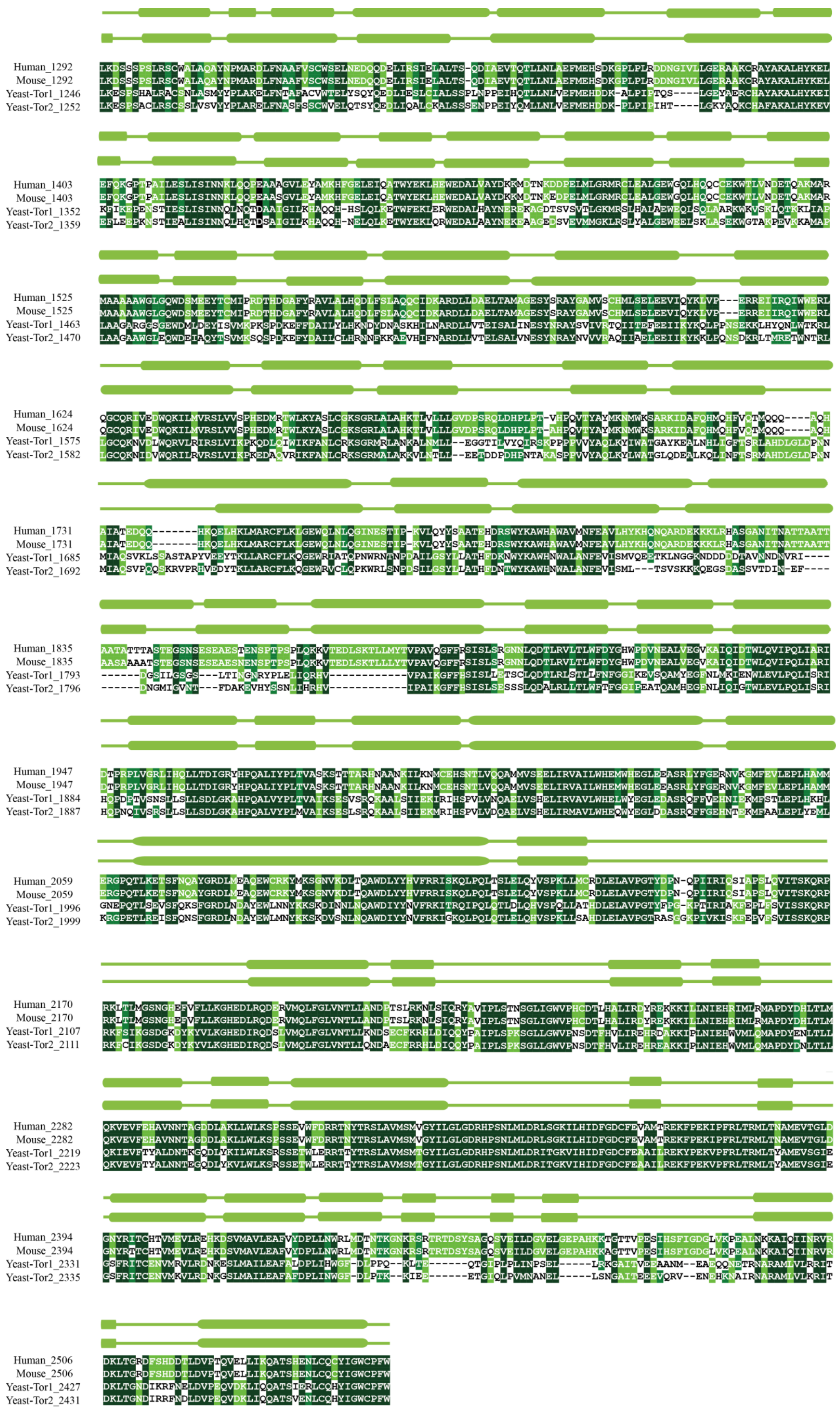
Human_1292
Mouse_1292
Yeast-Tor1_1246
Yeast-Tor2_1252
Human_1403
Mouse_1403
Yeast-Tor1_1352
Yeast-Tor2_1359
Human_1525
Mouse_1525
Yeast-Tor1_1463
Yeast-Tor2_1470
Human_1624
Mouse_1624
Yeast-Tor1_1575
Yeast-Tor2_1582
Human_1731
Mouse_1731
Yeast-Tor1_1685
Yeast-Tor2_1692
Human_1835
Mouse_1835
Yeast-Tor1_1793
Yeast-Tor2_1796
Human_1947
Mouse_1947
Yeast-Tor1_1884
Yeast-Tor2_1887
Human_2059
Mouse_2059
Yeast-Tor1_1996
Yeast-Tor2_1999
Human_2170
Mouse_2170
Yeast-Tor1_2107
Yeast-Tor2_2111
Human_2282
Mouse_2282
Yeast-Tor1_2219
Yeast-Tor2_2223
Human_2394
Mouse_2394
Yeast-Tor1_2331
Yeast-Tor2_2335
Human_2506
Mouse_2506
Yeast-Tor1_2427
Yeast-Tor2_2431

Figure S5: The sequence alignment used the sequence of mTOR from human. The upper is secondary structure from our resolved structure (yellow label means the sequence missed in our structure, we use predict result), the lower is predicted secondary structure from www.predictprotein.org. The numbers of each sequence is shown after the name in each line. N-HEAT and Core domain is shown in green, M-HEAT is shown in purple. Each sequence corresponding number is: Human, GI:1169735; Mouse, GI:227330586; TOR1-Yeast,GI:468739; TOR2-Yeast,GI:298028. The sequences were aligned and colored by Genedoc. The fully conserved residues are in white letters against deep green background, while scored as similar are represented by white letter on green and light green background.

Yang_Fig S6

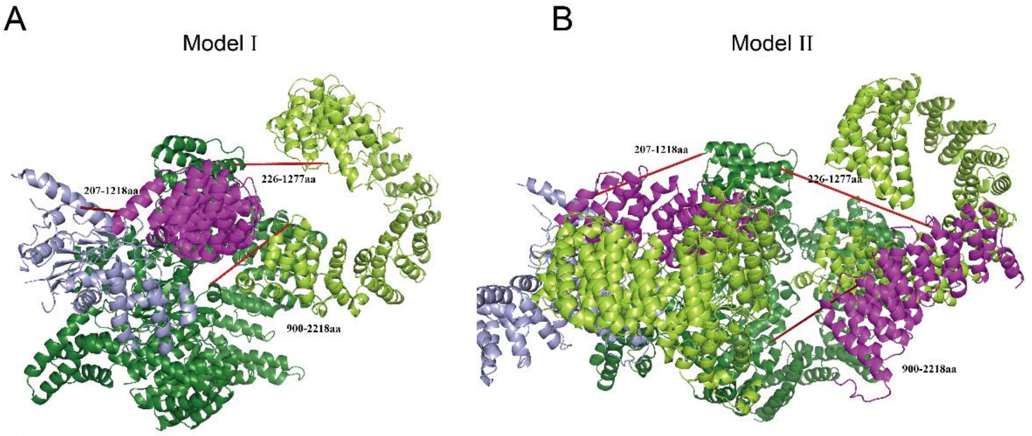

C

Residues in N-HEAT and Core region

Protein1-Protein2	#Spec-Total	Best E-value	**Model I** Distance (Å)	**Model II** Distance (Å)	**Model III** Distance (Å)	**Model IV** Distance (Å)
mTOR(226)-mTOR(1277)	6	5.75E-19	37	62	62	37
mTOR(898)-mTOR(2218)	10	8.45E-16	21	70	NDA	21
mTOR(900)-mTOR(2218)	14	2.05E-15	24	70	NDA	24
mTOR(230)-mTOR(1277)	2	2.97E-10	40	61	61	40
mTOR(353)-mTOR(1566)	3	4.88E-09	32	30	40	32
Mean distance			30.5	58.6	54.3	30.5

Residues in M-HEAT and Core, Core and Raptor

Protein1-Protein2	#Spec-Total	Best E-value	**Model I** Distance (Å)	**Model II** Distance (Å)	**Model III** Distance (Å)	**Model IV** Distance (Å)
mTOR(980)-mTOR(1257)	4	1.25E-17	28	66	36	28
mTOR(980)-mTOR(1256)	16	1.03E-11	28	66	36	27
mTOR(1218)-Raptor(207)	20	3.1E-11	28	55	34	55
Mean distance			28	62.3	35.3	36.7

Figure S6: (A-B) The key crossed linked amino acids in mTORC1 structure. The distance of three pairs of amino acids: mTOR(226)-mTOR(1277), mTOR(900)-mTOR(2218), mTOR(1218) - Raptor(207) are shown in the mTORC1 structure. The distance of model I (A) is shorter than model II (B). (C) The MS data facilitates structural identification of model I of mTORC1 topology in the EM density.

Yang_Fig S7

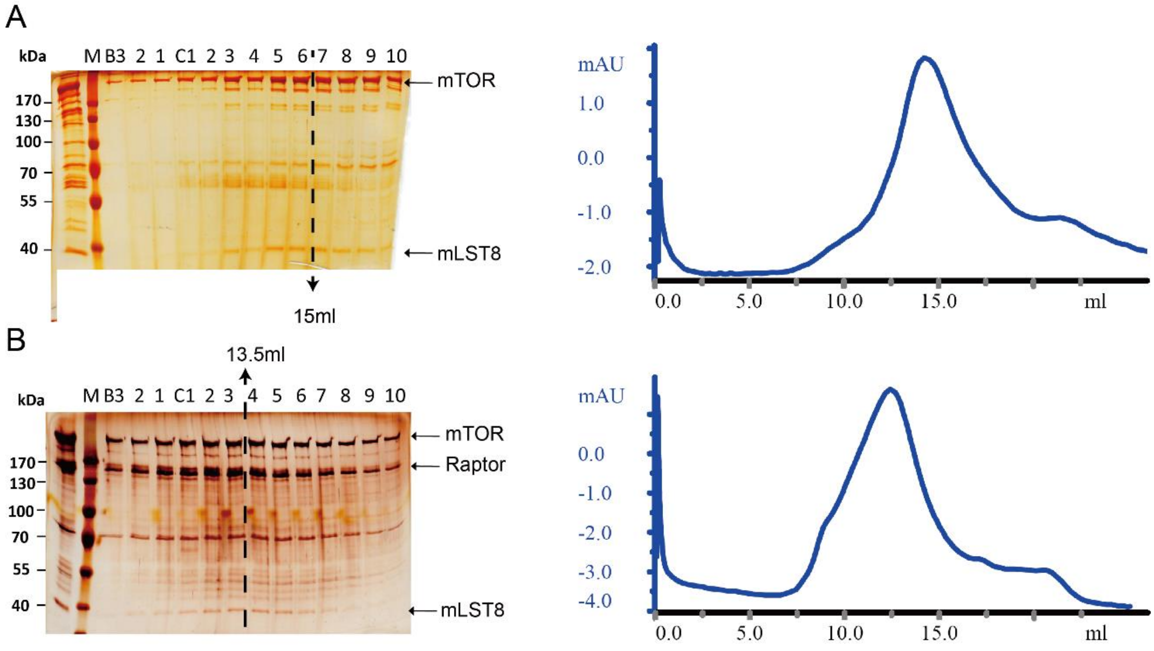

Figure S7: Dimerization of mTOR in solution. Equal amounts of mTOR-mLST8 (A) and mTOR-mLST8-Raptor (B) were applied to Superose 6 (right panel profile) and the peak fractions were subjected to SDS-PAGE for silver staining (left panel). The peak positions for the two complexes are indicated with dashed lines. About 1.5 ml migration difference was observed, suggesting that mTOR-mLST8 forms dimer in solution.
